# An Herbal Product Alleviates Bleomycin-Induced Pulmonary Fibrosis in Mice via Regulating NF-κB/TNF-α Signaling in Macrophages

**DOI:** 10.3389/fphar.2022.805432

**Published:** 2022-04-25

**Authors:** Fei Jing, Xi Chen, Jingbo Xue, Kai Huang, Feng Xing, Xudong Hu, Yuan Peng, Chenghai Liu

**Affiliations:** ^1^ Institute of Liver Diseases, Shuguang Hospital Affiliated to Shanghai University of Traditional Chinese Medicine, Shanghai, China; ^2^ Shanghai Key Laboratory of Traditional Chinese Clinical Medicine, Shanghai, China; ^3^ School of Basic Medical Sciences, Shanghai University of Traditional Chinese Medicine, Shanghai, China; ^4^ Key Laboratory of Liver and Kidney Diseases, Ministry of Education, Shanghai, China

**Keywords:** fuzheng huayu formula, pulmonary fibrosis, network pharmacology, macrophages, TNF-α signal pathway, NF-κB, lipopolysaccharide, methylprednisolone

## Abstract

**Background and aim:** Pro-inflammatory macrophages aggravated progress of pulmonary fibrosis (PF) both in patients and animal models. Fuzheng Huayu (FZHY) formula, a Chinese herbal product, is effective in treating pulmonary fibrosis in our previous study. But its action mechanism against PF relating to macrophage activation was unclear. This study was designed to evaluate the anti-fibrotic and anti-inflammatory roles of FZHY in pulmonary fibrosis and to elucidate the potential mechanisms.

**Methods:** Network pharmacology was employed to identify the interrelationships among compounds of FZHY, potential targets and putative pathways on anti-pulmonary fibrosis. According to the data of bioinformatics analysis, the key pharmacological target for FZHY against PF was screened. The network pharmacological prediction was validated by a series of experimental assays, including CCK8, western blot and immunofluorescence staining. Then molecular mechanism of FZHY on relating to the predictive target were studied in bleomycin induced pulmonary fibrosis in mice with methylprednisolone as a positive control, and in lipopolysaccharide (LPS) stimulated cultured macrophages in culture, respectively.

**Results:** The network pharmacology analysis reveal that a total of 12 FZHY–PF crossover proteins were filtered into a protein-protein interaction network complex and designated as the potential targets of FZHY against pulmonary fibrosis, while TNF-α signal pathway ranked at the top. FZHY and methylprednisolone could attenuate the lung fibrosis and decrease pulmonary TNF-α expression in bleomycin induced fibrotic mice, without difference between two treatments. While TNF-α was mainly originated from macrophages identified by double fluorescent staining of TNF-α and F4/80. LPS stimulated cultured macrophage polarization and activation demonstrated by the enhance contents of TNF-α and iNOS but decreased level of Arg-1. FZHY could alleviate the LPS stimulated macrophage polarization and activation demonstrated by decreasing TNF-α and iNOS and increasing Arg-1. In particular, FZHY could significantly reduce the production of p65 and the nuclear translocation of phosphorylated p65.

**Conclusion:** Fuzheng Huayu formula has a good effect against pulmonary fibrosis induced by bleomycin in mice, whose action mechanism was associated with down-regulation of NF-κB/TNF-α signaling pathway in pro-inflammatory macrophages. These findings provided an important strategy for developing new agents against lung fibrosis and accelerated FZHY product application on patients with lung fibrosis.

## Graphical Abstract

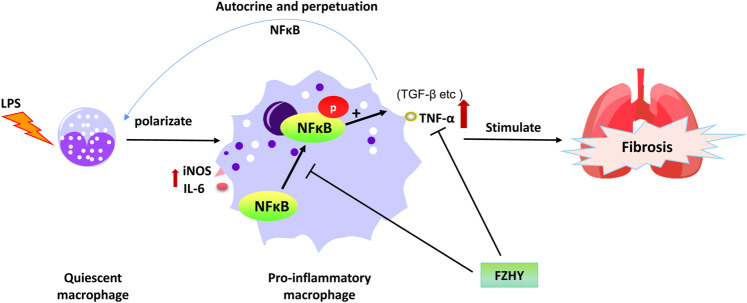

## Introduction

Pulmonary fibrosis (PF) is a chronic interstitial lung disease caused by various insults including chemical materials, smoke, microbial infection, etc (Noble et al., 2012). As a highly heterogeneous and lethal pathological process, pulmonary fibrosis is characteristic of collage deposition and architecture destruction in lung that ultimately induce organ malfunction, disruption of gas exchange, and death from respiratory failure (Wynn, 2011). There are very limited therapeutic options for PF, except lung transplantation, two antifibrotic drugs, Pirfenidone and nintedanib have been approved by FDA for PF treatment (King et al., 2014; Karimi-Shah and Chowdhury, 2015). But the therapeutic effects of these two agents were not completely satisfactory in clinic (Ahluwalia et al., 2014; Kingwell, 2014). In fact, pulmonary fibrosis has complicated processes involving multiple targets, refractory to be treated or cured with single targeted agent. Traditional Chinese Medicine (TCM) is characterized by multiple components and multiple targets, as well as rich experiences of treating chronic lung diseases. Therefore, the efforts to develop the anti-lung fibrotic agents from TCM could be a practical approach to meet the clinic emerging needs.

Fuzheng Huayu (FZHY) formula is a botanic product composed of six herbs (Table 1). The randomized controlled trial (RCT) studies in multi centers had approved that FZHY could regress liver fibrosis due to hepatitis B (Wang et al., 2018). It was approved as an herbal product for liver fibrosis treatment by China National Medical Products Administration (NMPA) since 2002, and clinic trails in US for liver fibrosis due to HCV has been carried out. The action mechanism of FZHY against liver fibrosis was integrative, involving in protection of hepatocyte inflammation, inhibition of stellate cell activation via TGF-β/Smads pathway, and regulating hepatic matrix metabolism etc. (Liu et al., 2009). And recently, we found that FZHY could regulate immunity in diseased liver (Zhang et al., 2020). In particular, FZHY was found to have favorable effects on pulmonary interstitial inflammation and fibrosis in Bleomycin induced rats in our previous study (Dai et al., 2006; Tan et al., 2007), and helpful in improving lung function for Chronic obstructive pulmonary disease (COPD) patients as well [Published in Chinese]. However, little is known about the mechanism of action for FZHY against lung fibrosis.

**TABLE 1:** Components of Fuzheng Huayu formula.

Chinese name	Plant sources	Medicinal parts	Preparation amount (g)
Danshen	Salvia Miltiorrhizae Bge (Labiatae)	radix	8
Chongcao	artificial fermentation cordyceps	mycelia	4
Taoren	Prunus persica (L.) Batsch (Rosaceae)	fruit	2
Jiaogulan	Gynostemma pentaphyllum (Thunb)	whole herb	6
Songhuafen	Pinus massoniana Lamb (Pinaceae)	pollen	2
Wuweizi	Schisandrae Chinensis (Turcz.)Baill	fruit	2

Pulmonary fibrosis is associated with inflammation characterized by the recruitment of macrophages, neutrophils and lymphocytes in the airways (Ward and Hunninghake, 1998). Among these, macrophage infiltration was the most crucial for regulating the progression of lung fibrosis (Wynn and Vannella, 2016; Yao et al., 2016).

Activation of macrophages could accelerate the recruitment and activation of other immune cells, thus further amplify the pulmonary inflammation, initiate and develop lung fibrosis. During this period, inflammatory mediators (eg. TNF-α) and pro-fibro-genic factors (eg. TGF-β1) were released, which accelerated the deterioration of the lung tissue, stimulated fibroblast activation. As a result, overproduction and deposition of ECM were accumulated in the lung tissue, and eventually fibrosis occurred. Could FZHY play its action against-lung fibrosis via anti-inflammation as it acted against liver fibrosis via protecting hepatocyte from injury and inflammation? Or are there any other mechanisms, through which FZHY plays a comprehensive role in preventing or regressing lung fibrosis?

**FIGURE 1:**
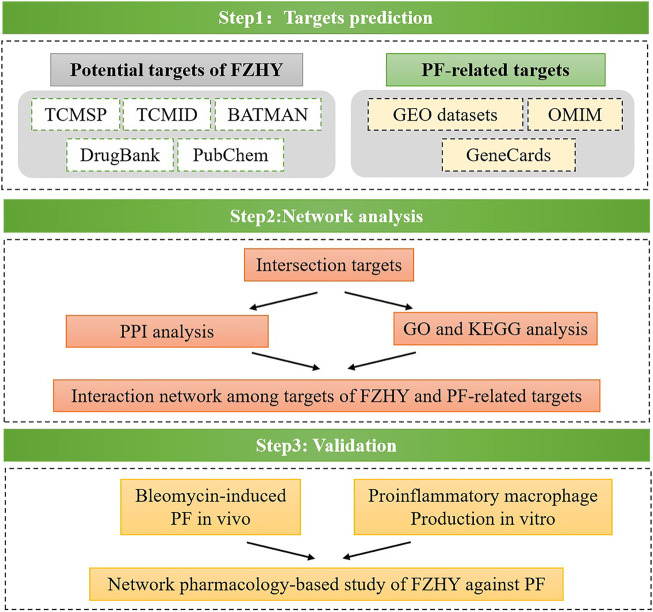
Flow chart.

By focusing on the above questions, we applied a network pharmacology approach with online database to search for probable target genes of FZHY related to pulmonary fibrosis treatment. Then a series of experimental assays were performed *in vivo* and *in vitro* to validate the effects of FZHY on pulmonary fibrosis and their underlying mechanism (Figure 1). With the above works, we predicted that TNF-α was the main target of FZHY against pulmonary fibrosis, which had been validated in Bleomycin induced lung fibrosis model in mice. Moreover, macrophage was the main resource of TNF-α in diseased lung, and we elucidated that FZHY could inhibit pro-inflammatory macrophage activation via NF-κB/TNF-α signaling pathway, which was closely associated with its efficacy on lung fibrosis. These results might represent a breakthrough in the potential use of FZHY for pulmonary fibrosis treatment.

## Materials and Methods

### Ethics

All experimental procedures complied with the Guidelines for Experimentation of Shuguang Hospital, affiliated with Shanghai University of Traditional Chinese Medicine. The protocols were reviewed and approved by the Ethics Committee of the institution.

### Databases

Data on the canonical name and molecular weight of the compounds in FZHY were obtained from the Traditional Chinese Medicine Systems Pharmacology database and Analysis Platform (TCMSP; http://tcmspw.com/tcmsp.php, version 2.3, updated on april 30, 2021 (Ru et al., 2014), the Bioinformatics Analysis Tool for Molecular Mechanism of Traditional Chinese Medicine (BATMAN-TCM; http://bionet.ncpsb.org/batman-tcm/, updated on april 30, 2021) (Liu et al., 2016), and PubChem (https://pubchem.ncbi.nlm.nih.gov, updated on april 1, 2021; (Kim et al., 2019). To identify the compounds with high levels of oral absorption, usability, and biological activity for further study, candidate components were screened and selected based on the following two parameters: 1) oral bioavailability (OB) of ≥30% and 2) drug-likeness (DL) of ≥0.18.

### Prediction of Drug-Related Targets of Bioactive Components

Predicted target genes of FZHY ingredients were screened in two online databases, TCMSP and BATMAN-TCM (Ru et al., 2014; Liu et al., 2016). The known therapeutic targets of these ingredients were confirmed by referring to DrugBank database (www.drugbank.ca, updated on 28 February 2020) and designated as the putative targets of FZHY. The putative targets were designated as the drug-related genes.

### Prediction of PF-Related Genes From Gene Expression Omnibus Profiles

Gene expression microarray data (GSE76808) on the gene expression profiles of PF in patients with and without systemic sclerosis-related interstitial lung disease were obtained from Gene Expression Omnibus (GEO) database of the National Center for Biotechnology Information (https://www.ncbi.nlm.nih.gov/geo), a public functional genomics data repository. Data of 18 lung samples were obtained, four of which from individuals without PF (GSM2038281, GSM2038282, GSM2038283 and GSM2038284) and 14 of which from patients with PF (GSM2038267, GSM2038268, GSM2038269, GSM2038270, GSM2038271, GSM2038272, GSM2038273, GSM2038274, GSM2038275, GSM2038276, GSM2038277, GSM2038278, GSM2038279 and GSM2038280). By using the data from the whole human genome microarray, we analyzed the potential therapeutic targets for patients with and without PF by employing the LIMMA package in the R language. An adjusted *p* value of <0.05 and a fold change (FC) of >1.2 were selected as the cutoff criteria. The potential genes were considered PF-related target genes and processed by using Strawberry Perl (version 5.30.0.1) and R (version 4.0.2) with the Bioconductor packages.

### Prediction of PF-Related Targets From Online Databases

Information regarding the various genes associated with PF were obtained from the GeneCards database (http://www.genecards.org) and Online Mendelian Inheritance in Man (OMIM) database (http://omim.org/), which are knowledge bases of human genes and genetic disorders (Hamosh et al., 2005; Stelzer et al., 2016). The key search terms used in two databases were “lung fibrosis” and “Pulmonary fibrosis” and the results were exported online.

### Processing of Data on Potential Hub Genes Involved in Effects of FZHY on PF

The predictions of PF-related genes and putative drug-related genes were verified by VennDiagram R package. Overlapping genes were considered as potential hub genes related to the preventative effects of FZHY against PF.

### Network Construction and Analysis

On the basis of the potential hub genes identified through the aforementioned process, a protein–protein interaction (PPI) network was established by public Search Tool for the Retrieval of Interacting Genes/Proteins database (https://string-db.org, updated 21 May 2021) (Szklarczyk et al., 2015). The minimum required interaction score was set to 0.7 to improve the accuracy of results. For further observations of biological functions of the hub genes, the cluster Profiler package was used to explore and visualize the data used in the pathway and functional enrichment analysis. Cytoscape (version 3.7.1) was used to construct and visualize a network of the compound–target protein-differential genes, which can be used to identify the target proteins that connect between compounds and differential genes in PF.

### Gene Ontology and Kyoto Encyclopedia of Genes and Genomes Pathway Analysis

To identify the functional annotation and pathway enrichment associated with the potential genes, a pathway enrichment analysis was performed by Gene Ontology (GO) annotation database (http://www.geneontology.org) and Kyoto Encyclopedia of Genes and Genomes (KEGG) (http://www.genome.jp/kegg) using cluster Profiler R package (Ashburner et al., 2000; Kanehisa et al., 2019). The enrich plot and DOSE Bioconductor packages were used to visualize the enrichment results and facilitate the interpretation process (Yu et al., 2015). Biological process (BP), molecular function (MF), and cellular component (CC) were used to visualize the functional annotation and pathway of potential genes. Enrichment with a *p* value of <0.05 was considered statistically significant, and an adjusted *p* value of <0.05 was set as the threshold value.

### Drug Preparation and Identification

FZHY formula was purchased from Shanghai Sundise Traditional Chinese Medicine Co., Ltd, and was prepared as previously described (Liu et al., 2019; Wu et al., 2019) Table 2.

**TABLE 2:** The chemical components of FZHY for FZHY quality control.

Compounds (marker)	Quality criterion
Salvianolic acid B (from Danshen)	Should be no less than 15.6 mg in 24 g of FZHY raw materials (daily dose)
Sodium Danshensu (from Danshen)	Should be no less than 13.2 mg in 24 g of FZHY raw materials (daily dose)
Adenosine (from Chongcao)	Should be no less than 4.8 mg in 24 g of FZHY raw materials (daily dose)
Schisandrin B (from Wuweizi)	Should be no less than 2.28 mg in 24 g of FZHY raw materials (daily dose)

### Mouse Models

Male C57BL/6 mice (24 ± 2 g) were supplied by Zhejiang Vital River Laboratory Animal Technology Co., Ltd. (License No: SCXK (Zhe)2019–0001). The mice were fed in the Experimental Animal Center of Shanghai University of Traditional Chinese Medicine and were acclimatized to the animal center conditions for 3 days before experiments. The mice were given Rodent Laboratory Chow and water ad libitum and maintained under controlled conditions with a temperature of 22–25°C, relative humidity of 46–52%, and a 12/12-h light/dark cycle (lights on at 7a.m.). Our experiments were conducted in accordance with the Animal Ethics Committee of Shanghai University of Traditional Chinese Medicine (License No: PZSHUTCM200821006).

### Bleomycin-Induced Murine Pulmonary Fibrosis Model

We utilized a modified model of bleomycin-induced pulmonary fibrosis according to our previous work (Tan et al., 2007). Briefly, Bleomycin (CSN10472, Csnpharm, USA) dissolved in saline was administrated via trachea route at a single dose of 2 mg/kg at day 0. The sham-treated mice were treated with the identical volume of normal saline.

### Animal Groups and Experimental Design

To evaluate the effect of FZHY on PF, 46 C57BL/6 mice were randomly divided into four treatment groups: normal control (n = 10), model control (n = 12), FZHY treatment (n = 12) and positive control (n = 12). Mice in model control, FZHY treatment and positive control groups were received bleomycin via trachea route to induce pulmonary fibrosis model at Day 0. In addition, for FZHY treatment groups, FZHY was administered daily through oral gavage at doses of 5.6 g/kg body weight once a day for 20 consecutive days. The daily amount of FZHY was equivalent to 10 times of the clinical dose of 60 kg/day body weight in adults. Mice in positive control group was intragastrically administered methylprednisolone (Pfizer Italia Srl, H20150245) at a daily dose of 10 mg/kg body weight. For the normal control and model control groups, the mice were treated with saline through gavage. All mice were sacrificed with pentobarbital sodium and killed 24 h after the last administration.

### Pulmonary Histopathology

Morphological evaluation was performed on formalin-fixed, paraffin-embedded lung tissue sections and stained with hematoxylin and eosin (HE) to investigate levels of lung inflammation in accordance with manufacturer’s procedures. For estimating the lung collagen deposition, Masson’s trichrome staining (collagen stains blue) and picrosirius red staining (collagen stains red) were performed. The images were analyzed using a light microscope (Olympus BX40, Japan).

### Immunohistochemical Analysis

Immunohistochemical analysis was performed to detect TNF-α and F4/80 expressions in lung tissues. In brief, paraffin-embedded sections were deparaffinized in xylene twice for 10 min and rehydrated in graded concentrations of ethanol (100%, 95%, 90%, 80% and 70%) and then submerged to water. For antigen retrieval, the whole sections were submerged in antigenic retrieval buffer and microwaved. They were then treated with 3% hydrogen peroxide in methanol to inactivate the endogenous enzymes. Sequentially, the sections were incubated with 5% bovine serum albumin (BSA) for 20 min in room temperature to block nonspecific binding. Sections were incubated with anti-TNFα (Absin, abs131997, China) or F4/80 (CST, 70076T, USA) overnight at 4°C, respectively. After washing with phosphate-buffered saline (PBS), tissue sections were treated with secondary antibodies, followed by incubation with conjugated horseradish peroxidase–streptavidin. Tissue sections were then counter-stained with hematoxylin, dehydrated and mounted.

### Immunofluorescence Staining

The lung tissue sections or cells were fixed with cold acetone for 30 min and treated with 0.5% TritonX-100 for 2 min. Then they were blocked with 1% bovine serum albumin for 40 min, and stained with primary antibody. After washing, they were incubated with fluorescent secondary antibody. Nuclei were stained with 4′, 6-diamidino-2-phenylindole (DAPI; ab228549, abcam, USA). Images and photographs were obtained by Olympus confocal microscope.

### Isolation of Murine Bone Marrow-Derived Macrophages

10–12 weeks old male C57BL/6 mice were euthanized. Subsequently, the knee joint was cut and both ends of tibia and femur were removed one by one. The bones were washed with sterile PBS. The ends of the bones were removed to flush out the bone marrow with DMEM containing penicillin-streptomycin. Next, the bone marrow was resuspended and passed through a 70 μm nylon filter to remove debris and unwanted tissue. Cells were centrifuged for 10 min at 1,500 rpm. The cell pellet was resuspended in 3 ml red blood cell (RBC) lysis buffer. RBCs were lysed for 2 min at room temperature (RT). Cells were separated by centrifugation (10 min, RT, 1,500 rpm). RBC-free cell pellets were resuspended in DMEM containing of 10% FBS without penicillin-streptomycin, and cells were enumerated by using a TC-20 cell counter (Biorad, USA). Cells were adjusted to 3.0 × 10^5^ cells/ml in BMDM medium and seeded into T150 flask (NEST, China). Medium was changed 4 days later. After 7 days of culture, 99% of the attached cells were BMDMs.

### Cell Viability Assay

CCK8 cell viability kit (HY-K0301, MCE, China) was used to detect cell viability. Briefly, BMDMs were seeded in 96-well plates (3×10^4^ cells/well) and 100 μl of 10% CCK8 solution were added into each well, followed by incubation in a humidified incubator for 2 h at 37°C, 5% CO_2_. After the incubation, absorbance was measured at 450 nm and 630 nm using a microplate reader (BioTEK, MQX200R, USA).

### Cytokine Quantification

TNF-α (227846-011, Thermofisher, Austria) and IL-6 (234277-008, Thermofisher, Austria) released in supernatants were quantified by ELISA according to manufacturer’s protocol.

### Western Blot Analysis

Cell pellets or lung tissues were lysed with RIPA buffer containing a complete cocktail of protease inhibitors in western blot analysis (53153900, Roche, Germany). Proteins were quantified by BCA Protein Assay (WA322434, Thermofisher, USA) and equal amounts (20 μg) of total protein lysates from each sample were separated by SDS-polyacrylamide gel electrophoresis (SDS-PAGE). Separated proteins were then transferred onto polyvinylidene fluoride (PVDF) or nitrocellulose (NC) membranes. Membranes were blocked with blocking buffer (WB315798, Thermofisher,USA) and then incubated with the following primary antibodies respectively: anti-TNF-α (1/1,000; 11948T, CST,USA), anti-Arg1 (1/1,000; 93668S, CST, USA), anti-iNos(1/1,000; 13120S, CST, USA), anti- NF-κB (1/1,000; 8242S, CST, USA), anti-p-NF-κB (1/1,000; 3033S, CST, USA), and anti-GAPDH (1/5000; 60004-1-Ig, Proteintech, China). Membranes were then thoroughly rinsed and incubated with species-matched HRP-conjugated secondary antibodies. Protein bands were visualized by ECL Western blot detection reagents (4600SF, Tanon, China) in accordance with manufacturer’s protocol or defined by scanning (Odyssey, USA) and quantified by computer-assisted image analysis system.

### Real-Time Fluorescence Quantitative Polymerase Chain Reaction

Total RNA was extracted using TRIzol Reagent (Sangon Biotech, Shanghai, China) in accordance with manufacturer’s protocol. After the concentration of RNA was determined, 500 ng of total RNA was used as the template for reverse transcription into single-stranded cDNA by Reverse Transcription Reagent Kit with gDNA Eraser (Takara, Dalian, China). Real-time fluorescence quantitative polymerase chain reaction was performed using SYBR Premix Ex Taq (Tli RNaseH Plus; Takara) and ViiA seven Real-Time PCR System (ABI, Carlsbad, CA, USA). The β-actin gene was amplified as an internal control, and the primer sequences are listed in Table 3. The relative gene quantities compared with β-actin were calculated through the 2^-∆∆CT^ method.

**TABLE 3:** Real-time quantitative PCR primers used in this study.

Gene	Forward (5’→3′)	Reverse (5’→3′)
TNF-α	TAG CCA GGA GGG AGA ACA GA	CCA GTG AGT GAA AGG GAC AGA
β-actin	TGA CGA GGC CCA GAG CAA GA	ATG GGC ACA GTG TGG GTG AC

### Statistical Analysis

All data were analyzed using PASW Statistics software (version 20). Differences between groups were assessed through a nonparametric one-way analysis of variance. Values were presented as mean ± standard deviation. *p* < 0.05 was considered statistically significant.

## Results

### Putative Ingredients and Targets for FZHY

We selected a total of 141 ingredients based on the following criteria for the absorption, distribution, metabolism, and excretion (ADME) properties of drugs with potential biological effects at a systematic level: OB ≥ 30% and DL ≥ 0.18 (Supplementary Table S1). The potential targets of FZHY were predicted by referring to the TCMSP, BATMAN-TCM and DrugBank databases, as described in the Materials and Methods section. A total of 197 potential target genes for FZHY were identified (Supplementary Table S2) after the redundant data were removed.

### Differentially Expressed mRNAs in Patients With and Without PF

**FIGURE 2:**
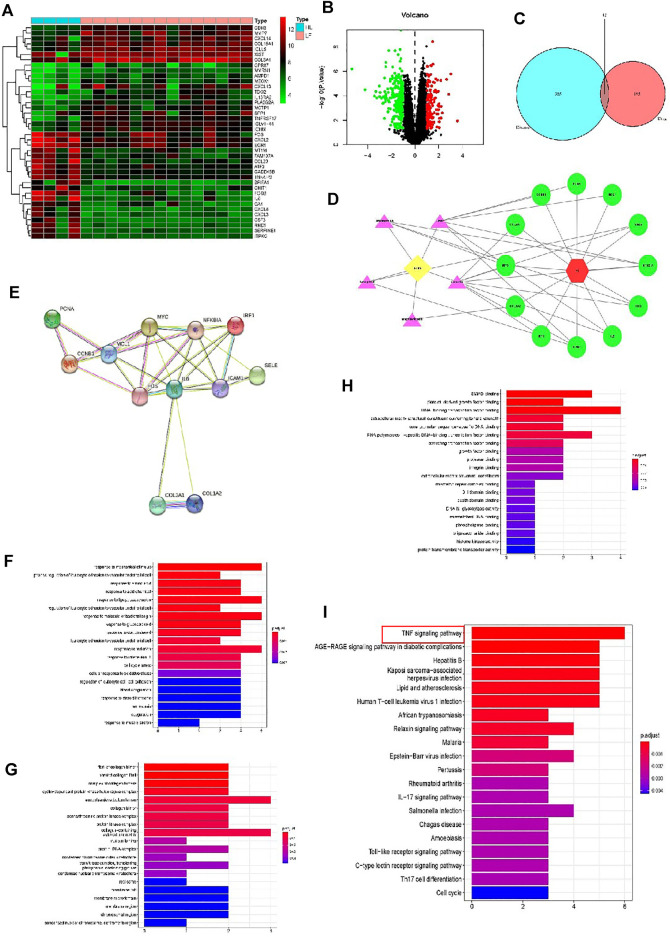
Analyses of the different potential therapeutic targets between PF and healthy lung tissues. **(A)** The heatmap comparing the different gene expressions between PF and healthy lung tissues was shown. **(B)** The volcano plot of *p* values as a function of weighted fold change for mRNAs in PF and healthy lung tissues. The vertical dotted line delimits up- and down-regulation. Red and green plots represent significant up-regulated and down-regulated mRNAs with>1.2-fold change and corrected *p* < 0.05, respectively. **(C)** Venn diagram of FZHY- and PF-related proteins. The overlapped genes were considered as the potential hub genes of FZHY against PF. **(D)** The Component-Target protein network. The violet triangles represent the candidate active compounds in FZHY. The green circles represent the gene names of target proteins of PF. **(E)** Cluster analysis of the PPI network. 12 FZHY-PF crossover proteins were filtered into the PPI network complex. **(F–H)** Bioinformatic analyses of drug-disease intersection proteins. Gene ontology annotations, including BP **(F)**, CC **(G)** and MF **(H)** analysis. **(I)** KEGG pathway enrichment analysis of 12 putative targets. **(I)** PF-related KEGG pathway enrichment was presented.

By using the raw data from the GSE76808 dataset, we analyzed mRNA expression profiles in the lung samples from patients with and without PF. Figure 2A presents a heatmap of the expression of the top 40 genes in the lung samples from the patients with PF and the controls. Volcano plot filtering identified 397 differentially expressed mRNAs (*p* < 0.05, |FC| > 1.2, Figure 2B). Among them, 158 mRNAs were upregulated, and 239 mRNAs were downregulated. Supplementary Table S3 presented the detailed information regarding these therapeutic targets used in the data analysis. In addition, the PF-related targets were predicted using the GeneCards and OMIM online databases (Supplementary Tables S4, S5).

### Construction of FZHY Target-PF Network

Based on the identified FZHY- and PF-related target genes, a Venn diagram of the FZHY- and PF-related proteins was created. The overlapping genes were considered differentially expressed genes (DEGs) and designated as potential hub genes for the effects of FZHY on PF (Figure 2C). A total of 12 DEGs were filtered into the PPI network complex and presumed to be related to the effects of FZHY on PF (Figure 2D; Table 4). In addition, a PPI network of the 12 DEGs was constructed (Figure 2E).

**TABLE 4:** The potential hub genes of FZHY against PF.

Gene	Full name
ICAM1	Intercellular Adhesion Molecule 1
IL6	Interleukin 6
FOS	Fos Proto-Oncogene
NFKBIA	Nuclear factor Kappa B inhibitor alpha
SELE	Selectin E
MCL1	Myeloid cell leukemia 1 protein
PCNA	Proliferating Cell Nuclear Antigen
CCNB1	cyclin B1
COL3A1	Collagen Type III Alpha 1 Chain
MYC	MYC proto-oncogene, bHLH transcription factor
COL1A2	Collagen Type I Alpha 2 Chain
IRF1	Interferon Regulatory Factor 1

To identify the relevant gene functions, the GO annotation, consisting of the BP, CC, and MF categories, displayed in Figures 2F–H, was assayed for the 12 DEGs by using R software. After KEGG analysis, DEGs were mapped to 53 KEGG pathways (Figure 2I and Supplementary Table S6). Notably, the top one ranked KEGG pathway was TNF signal pathway.

### FZHY Ameliorated Bleomycin-Induced Lung Fibrosis

**FIGURE 3:**
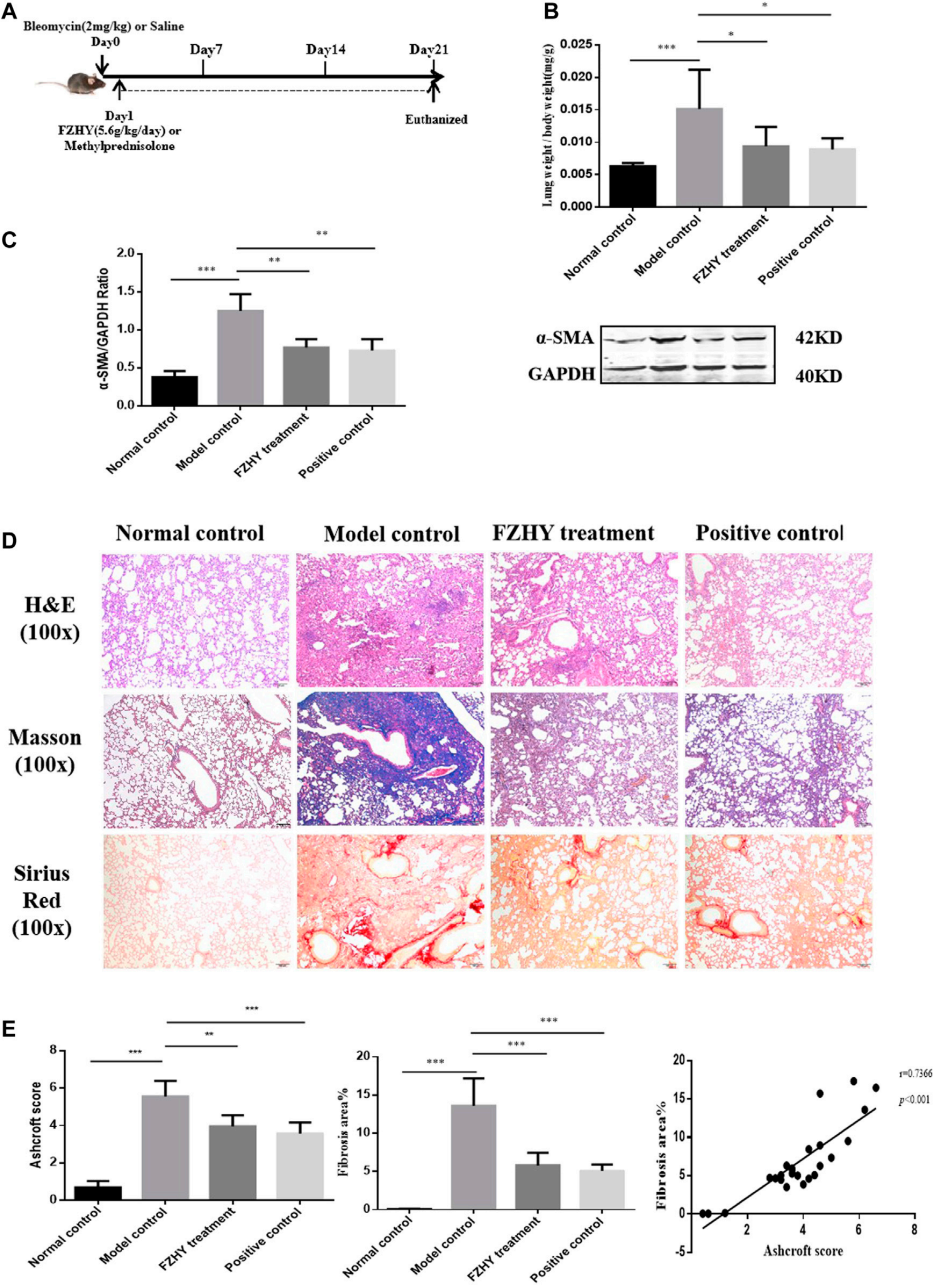
FZHY treatment attenuates bleomycin-induced lung fibrosis *in vivo*. **(A)** Lung fibrosis was induced by bleomycin at day 0. Then mice were administered with FZHY or methylprednisolone once a day for 20 consecutive days. 24 h after FZHY administration, all mice were sacrificed. **(B)** Ratio of lung weight to body weight in each group. **(C)** α-SMA protein expression in each group. **(D)** Pathological analysis including H&E, Masson, Sirius red stains at ×100 magnification in each tissue specimen. **(E)** Semi-quantitative analysis for collagen deposition. *p < 0.05; **p < 0.01; ***p < 0.001.

The pulmonary fibrotic mice were intervened with FZHY and methylprednisolone as positive drug respectively (Figure 3A). Since the intratracheal instillation of bleomycin in mice results in lung injury, which peaks 3–5 days later, and was followed by pulmonary fibrosis at 21 days (Chua et al., 2005; Budinger et al., 2006), the efficacy of FZHY and methylprednisolone on bleomycin-induced lung fibrosis in mice was evaluated 21 days after the administration. Compared with the normal mice, the lung weight was increased in model control (Figure 3B). FZHY and methylprednisolone treatment significantly decreased the lung weight compared to the model control (Figure 3B). The collagen deposition in lungs evaluated by Masson and Sirius red staining, and fibroblast activation measured as α-SMA expression with western blot (Figure 3C), was significantly increased in model control mice, but were decreased in FZHY and methylprednisolone groups compared to the model control. Also pulmonary inflammation appeared to also be more severe after bleomycin challenge, while FZHY treatment alleviated the inflammatory response in lung tissues (Figure 3D). Besides, the semi-quantitation for lung fibrosis with a numerical fibrotic scale was conducted (*Ashcroft score*) (Figure 3E). In, similar tendency in effects of FZHY against bleomycin-induced pulmonary fibrosis was observed, and there is no difference for the lung weight, fibrotic areas and α-SMA expression between FZHY and methylprednisolone. The results revealed that bleomycin-treated mice exhibited the increase of inflammation and collagen deposition, while FZHY treatment could suppress the changes caused by bleomycin.

### FZHY Reduced Expression of TNF-α in Macrophages in Mice Pulmonary Fibrosis *in vivo*

**FIGURE 4:**
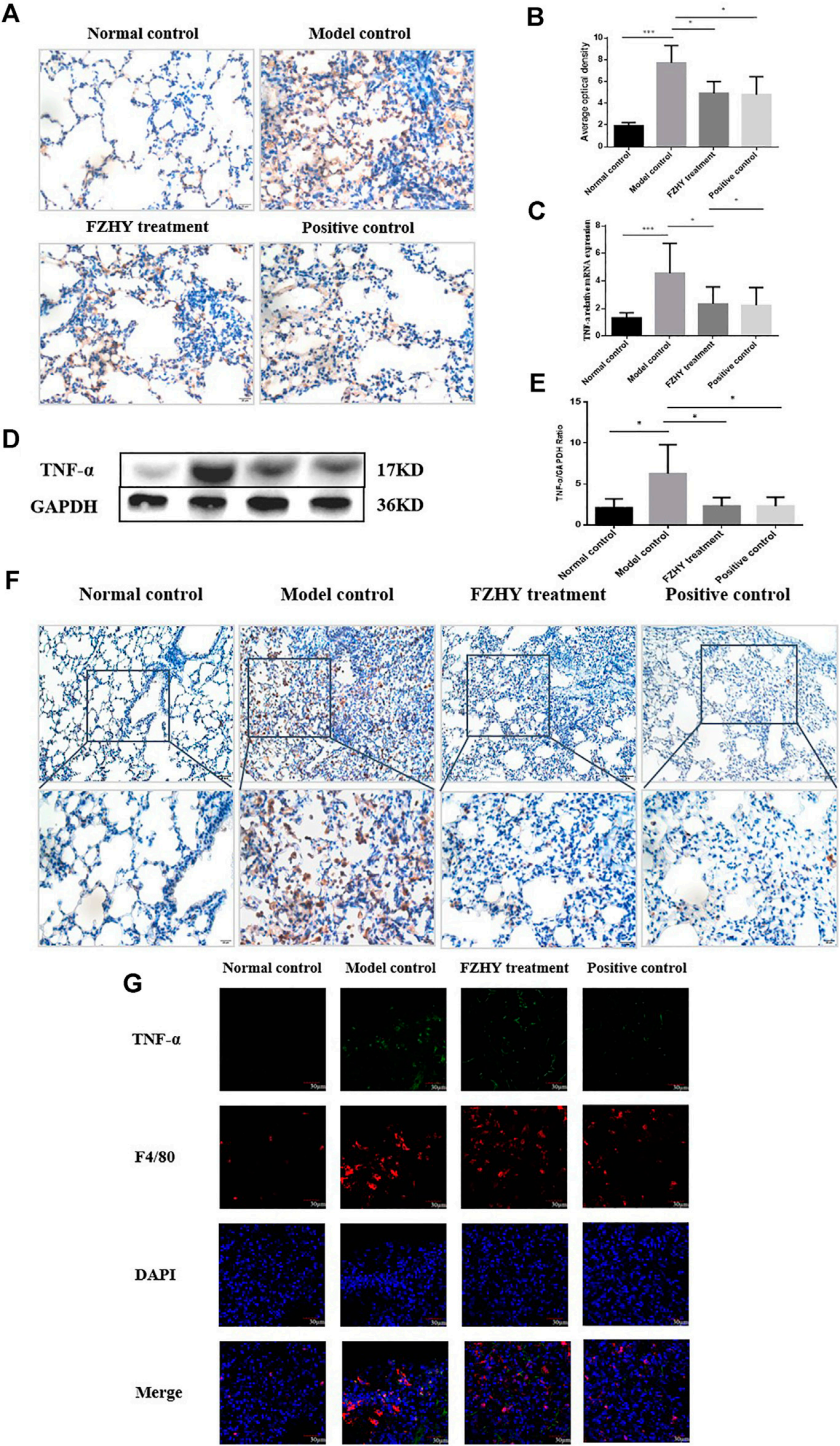
FZHY reduced expression of TNF-α in macrophages in mice Pulmonary Fibrosis *in vivo* **(A–B)** Immunohistochemistry for expression of TNF-α in the lung tissue (A; magnification, ×400) and semi-quantitative analysis for the positive-stain of TNF-α **(B)**. **(C)** mRNA expression of TNF-α in the lung tissue. **(D–E)** Western blot for TNF-α of the lung tissue **(D)** and histograms for Western blot results **(E)**. **(F)** Immunohistochemistry for expression of F4/80 in the lung tissue. **(G)** Representative immunofluorescence confocal microscopy images of F4/80 and TNF-α; red transfected into F4/80; green transfected into TNF-α. *p < 0.05; **p < 0.01; ***p < 0.001.

TNF-α expression in lung tissue was measured by immunohistochemical staining and western-blot assay. As shown in Figures 4A–E, positive staining, protein and gene (mRNA) expression of TNF-α were markedly increased in model control group than that in the normal mice. In particular, most of the positive staining of TNF-α located in lung interstitial area. Compared with the model group, TNF-α expression was remarkably decreased after FZHY or methylprednisolone as positive drug, but there is no significant difference between two treatment groups. To confirm whether expression of TNF-α was contributed by the inflammatory macrophages, we conducted double stains with antibodies of F4/80 [a mouse macrophage surface marker, (Austyn and Gordon, 1981; Lee et al., 1985)]. As Figures 4F-G showed that there were obvious increased positive stains for the single F4/80, and both F4/80 and TNF-α, i.e. inflammatory macrophages in model control group, compared to the normal mice, but those positive stains were decreased in FZHY or methylprednisolone treatment without difference between two treatments. These data revealed that FZHY treatment could significantly inhibited the aggregation of inflammatory macrophages in the lung.

### FZHY Downregulated TNF-α Expression via NF-κB Pathway in LPS-Stimulated BMDMs

**FIGURE 5:**
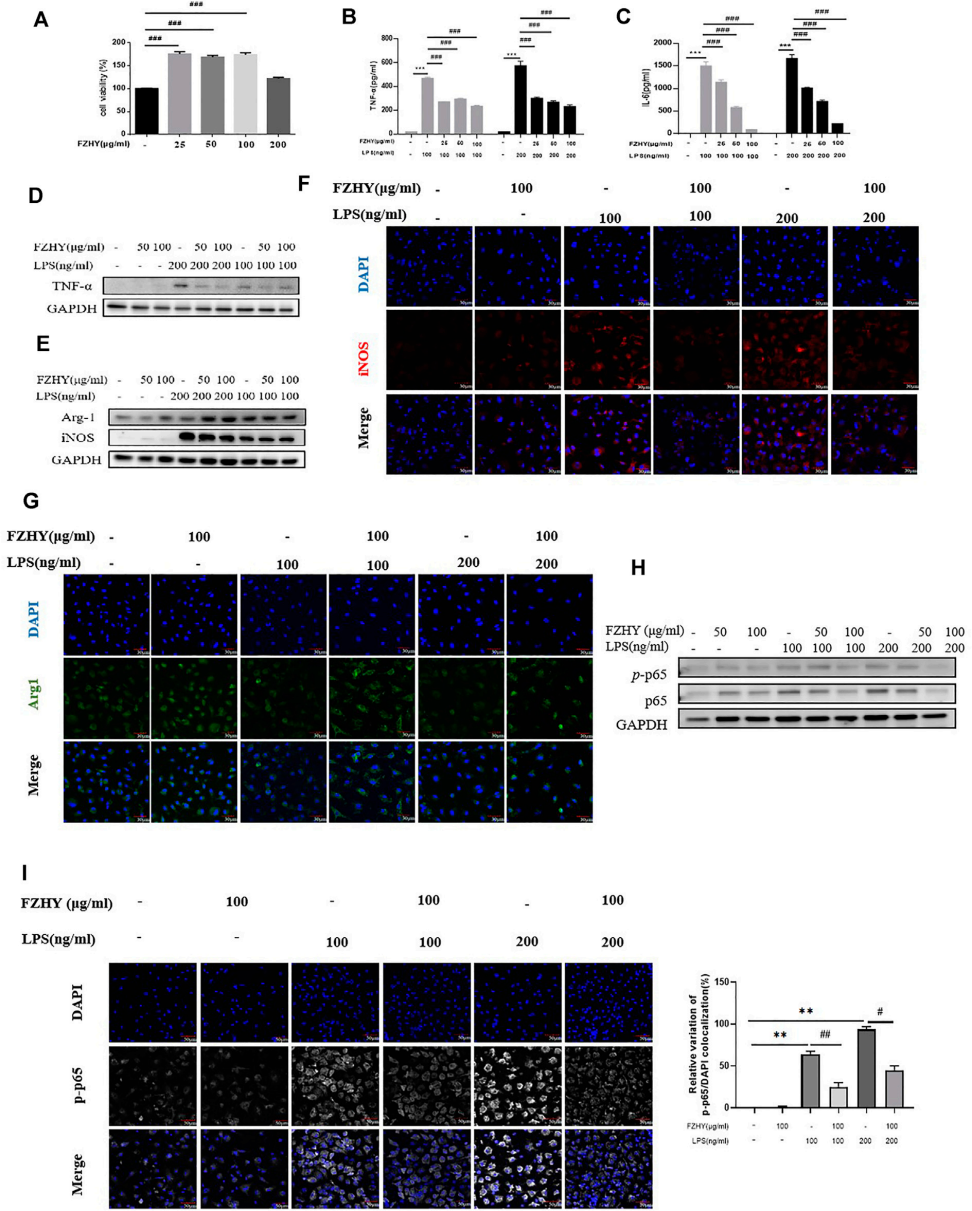
FZHY reduced the expression of TNF-α in LPS-inducedmacrophages in vitro. (A) Viability ofBMDMsat 24 h incubatedwith different concentrations of FZHY assessed byCCK8assay.BMDMswere treated withLPS (100 or 200 ng/ml) with or without FZHY(25 μg/ml, 50 μg/ml, 100 μg/ml) for 24 h (B–C) Levels of TNF-α and IL-6 in supernatant after 24 h incubated with different concentrations of FZHY observed by Elisa kits. (D) Western blot analysis for TNF-α, (E) Arg1 and iNos of the LPS-stimulated BMDMs. Representative immunofluorescence microscopy images of iNos (F) and Arg1 (G). (H) Western blot analysis for p65 and p-p65 production in the LPS-stimulated BMDMs.(I) Representative images of immunofluorescence staining of p-p65 NF-κB (white) nuclear translocation. Cell nuclei are detected by 4',6-diamidino-2-phenylindole (DAPI) (blue). Scale bars, 50 μm. Semi-quantitative evaluation of p-p65NF-κB nuclear translocation, as evidenced by the colocalization experiments.Results are expressed asmeans ± SDof the relative percentage of p-p65 nuclei-positively stained cells to the total number of cells. Ten fields per condition were analyzed. *p < 0.05, **p < 0.01, ***p < 0.001; #p < 0.05, ##p < 0.01, ###p < 0.001.

To elucidate the regulatory mechanism of FZHY on TNF-α expression in macrophages, we investigated the anti-inflammatory effects of FZHY on LPS-stimulated BMDMs. Firstly, BMDMs were incubated with 25–200 μg/ml of FZHY for 24 h, and there was no obvious cell toxicity during this dose scope. Secondly, BMDMs were exposed with 100 ng/ml and 200 ng/ml of LPS to induce proinflammatory polarization *in vitro, respectively*. Subsequently, cells were incubated with 25–100 μg/ml of FZHY for 24 h, the contents of TNF-α and IL-6 in cell supernatant were significantly inhibited by FZHY with a dose dependent manner (Figures 5B,C), which revealed that FZHY might be able to act a vital role in resisting inflammation by M1 macrophage polarization *in vitro*.

To confirm the anti-inflammatory effect of FZHY *in vitro*, we checked TNF-α expression with by western blot, the results showed that FZHY incubation for 24 h decreased TNF-α expression in LPS stimulated BMDM (Figure 5D; Supplementary Figure S5D). Besides, FZHY decreased the levels of iNos and enhanced Arg-1 (Figure 5E; Supplementary Figure S5E), respectively. In addition, compared with those in the LPS-simulated BMDMs (Figures 5F,G), immunofluorescence stains of Arg-1 and iNos were exhibited with the same tendency after FZHY incubation.

In further analysis, expression of component (p65) of NF-κB, the key transcription factor of TNF-α signal pathway, was significantly increased after LPS stimulation at a dose dependent manner. In contrast, levels of NF-κB components in pro-inflammatory BMDMs were effectively down-regulated in Figures 5H,I; Supplementary Figure S5H. The NF-κB nuclear translocation was moderately blocked after incubation of FZHY, which was demonstrated by the positive nuclear immunostaining (Figures 5H,I; Supplementary Figure S5H). Taken together, these results implicated that FZHY could inhibit activation of proinflammatory macrophages through NF-κB/TNF-α pathway.

## Discussion

Pulmonary fibrosis is characterized by an excess accumulation of collagen deposition in the lung tissue, accompanied by progressive dyspnea and worsening of pulmonary function with a high mortality rate. It has highly heterogeneous etiologies and complicated pathologic mechanisms. Although fibroblasts activation and differentiation played a pivot role in lung fibrogenesis, inflammatory mediators such as TNF and IL-1β have an important effect on the initiation and development of lung fibrosis. Macrophages and many other cells produce TNF after exposure to silica and bleomycin etc. While, methylprednisolone, the most used drug to inhibit inflammatory diseases in clinic, was effective for some type of lung fibrosis (Wynn, 2011). In current study, we firstly found that TNF-α related pathway could be the target of FZHY against lung fibrosis with informatic analysis, then FZHY was confirmed to inhibit the development of lung fibrosis in bleomycin-induced mice model, whereas methylprednisolone used as a positive control. Lastly, it revealed that the action mechanism of FZHY against lung fibrosis was associated with NF-κB/TNF-α signaling in LPS-stimulated macrophage.

Lung fibrosis is an orchestra process involving integrating mechanisms. TCM or herbal medicines comprise various active components, which could produce synergistic or antagonistic interactions among components through its multicomponent and multitarget principles (Wang et al., 2014). It is difficult to understand and uncover the main action mechanism of TCM due to its complexity. While a network pharmacology approach has the characteristic of integration and entirety, which is a very suitable tool to predict the target profiles and pharmacological mechanisms of active substances in TCM (Li and Zhang, 2013). FZHY formula, a traditional Chinese medicine formula, is used to treat liver fibrosis and cirrhosis in China. FZHY has been shown to have no serious adverse reactions and exerts antifibrotic effects through targeting multiple molecules (Chen et al., 2019). In our previous studies, FZHY could protect lung injury and fibrosis induced by bleomycin in rats, and improve pulmonary function in patients with COPD [Published in Chinese]. Herein, we applied the network pharmacology method to predict the mechanism of FZHY on lung fibrosis development. Given FZHY and other TCM products were taken orally, OB (Xu et al., 2012) and DL (Walters and Murcko, 2002), two ADME-related models, are the main factors affecting the absorption of drugs in gastrointestinal tract. Thus, we screened the bioactive components of FZHY by using two parameters: OB ≥ 30% and DL ≥ 0.18. A total of 197 active components were identified as FZHY-related genes, and 12 Differentially Expressed Genes (DEGs) were identified by bioinformatical analysis. To understand the relevant gene functions and the possible pathways of 12 DEGs, GO and KEGG pathway enrichment was analyzed through R software. The results revealed that TNF signaling pathway was markedly ranked first for FZHY action mechanism against lung fibrosis.

Studies have shown that TNF was a prerequisite for the development of pulmonary fibrosis, and TNF^−/−^ mice were completely unaffected by inflammation and fibrosis. The expression of TNF could not only trigger the initial acute inflammatory response to bleomycin-induced lung tissue injury, but also provide chronic inflammatory signals and lead to the progress of pulmonary fibrosis (Oikonomou et al., 2006). Therefore, the treatment strategy of inhibiting TNF production is of great significance in the treatment of pulmonary fibrosis. Based on the analysis results of network pharmacology, we further verified the anti-pulmonary fibrosis mechanism of FZHY by inhibiting TNF pathway *in vivo* and *in vitro*. Inflammatory macrophages are the main source of TNF(Shapouri-Moghaddam et al., 2018). In our animal experiment, the histological results showed that inflammation and coagulative necrosis were pronounced in the bleomycin-treated mice, while FZHY and methylprednisolone reduced lung inflammation and collagen deposition *in vivo*. Given inflammation is closely associated with the infiltration of macrophages in the lung, expressions of TNF-α and F4/80, a gold marker for macrophage, were observed to locate the origin of cells that mainly expressed TNF-α. The double staining result showed that macrophage is the main resource of TNF-α and increased in bleomycin-induced pulmonary fibrosis in mice. Meanwhile, FZHY and methylprednisolone decreased TNF-α^+^F4/80^+^ cell staining. It indicated that FZHY might inhibit experimental pulmonary fibrosis via down-regulate the activation of pro-inflammatory macrophages expressing TNF-α. In the *in-vitro* study, we revealed that FZHY inhibited pro-inflammatory macrophages activation, demonstrated by a decrease in release of TNF-α, IL-6 and iNOS, but increase of Arg1 in LPS-induced BMDMs. These above results suggested that FZHY might play an anti-pulmonary fibrosis role by inhibiting the inflammatory polarization of macrophages and reducing the production of TNF.

The most potent stimulus for eliciting TNF production by macrophage is LPS(Shapouri-Moghaddam et al., 2018), which could promote TNF production via NF-kB pathway (Tang et al., 2021). Therefore, blockade of NF-kB pathway is crucial step to inhibit macrophage activation and improve inflammation. NF-κB phosphorylation is a hallmark of NF-κB function to activate NF-κB-dependent transcription (Chen and Ivashkiv, 2010). p65 is one of the important subunits of NF-κB. Inhibiting p65 activation, that is, inhibiting the production of p-p65, can reduce LPS-induced acute lung injury and reduce the production of TNF(Yang et al., 2021). In the study, we observed NF-κB nuclear translocation in LPS-stimulated activation of pro-inflammatory macrophages with confocal immunofluorescence, and found that FZHY could significantly reduce the production and nuclear translocation of p65, indicating that FZHY could inhibit macrophage activation and inflammation via NF-κB/TNF-α pathway.

Our work indicates that macrophage is the important target cell for the therapy of pulmonary fibrosis, which was also proposed in liver fibrosis (Tacke, 2017). However, whether FZHY could influence lung fibroblasts directly via NF-κB/TNF-α pathway? What is the anti-pulmonary fibrosis molecular target in NF-κB/TNF-α pathway for the active components of FZHY? The answers to these questions would be our next research topic.

## Conclusion

FZHY was effective in alleviating pulmonary fibrosis, whose action mechanism was associated with regulation of NF-κB/TNF-α signaling pathway in pro-inflammatory macrophages. These findings provided an important strategy for developing new agents against lung fibrosis and accelerated FZHY product application on patients with lung fibrosis.

## Data Availability

The datasets presented in this study can be found in online repositories. The names of the repository/repositories and accession number(s) can be found in the article/Supplementary Material.

## Abbreviations

PF, pulmonary fibrosis; TCM, Traditional Chinese Medicine; FZHY, Fuzheng Huayu; BMDM, bone marrow-derived macrophages; LPS, lipopolysaccharide; RCT, randomized controlled trial; NMPA, National Medical Products Administration; COPD, chronic obstructive pulmonary disease; TCMSP, Traditional Chinese Medicine Systems Pharmacology database and Analysis Platform; BATMAN-TCM, Bioinformatics Analysis Tool for Molecular Mechanism of Traditional Chinese Medicine; GEO, Gene Expression Omnibus; OMIM, Online Mendelian Inheritance in Man; GO, Gene Ontology; KEGG, Kyoto Encyclopedia of Genes and Genomes; PVDF, polyvinylidene fluoride; PPI, protein–protein interaction; PBS, phosphate-buffered saline; NC, nitrocellulose; SDS-PAGE, SDS-polyacrylamide gel electrophoresis; DEGs, differentially expressed genes.
