# Supplementary figures and images for: An Herbal Product Alleviates Bleomycin-Induced Pulmonary Fibrosis in Mice via Regulating NF-κB/TNF-α Signaling in Macrophages
Source: Front Pharmacol. 2022 Apr 25;13:805432. doi: 10.3389/fphar.2022.805432 (PMC9081434; doi:10.3389/fphar.2022.805432)

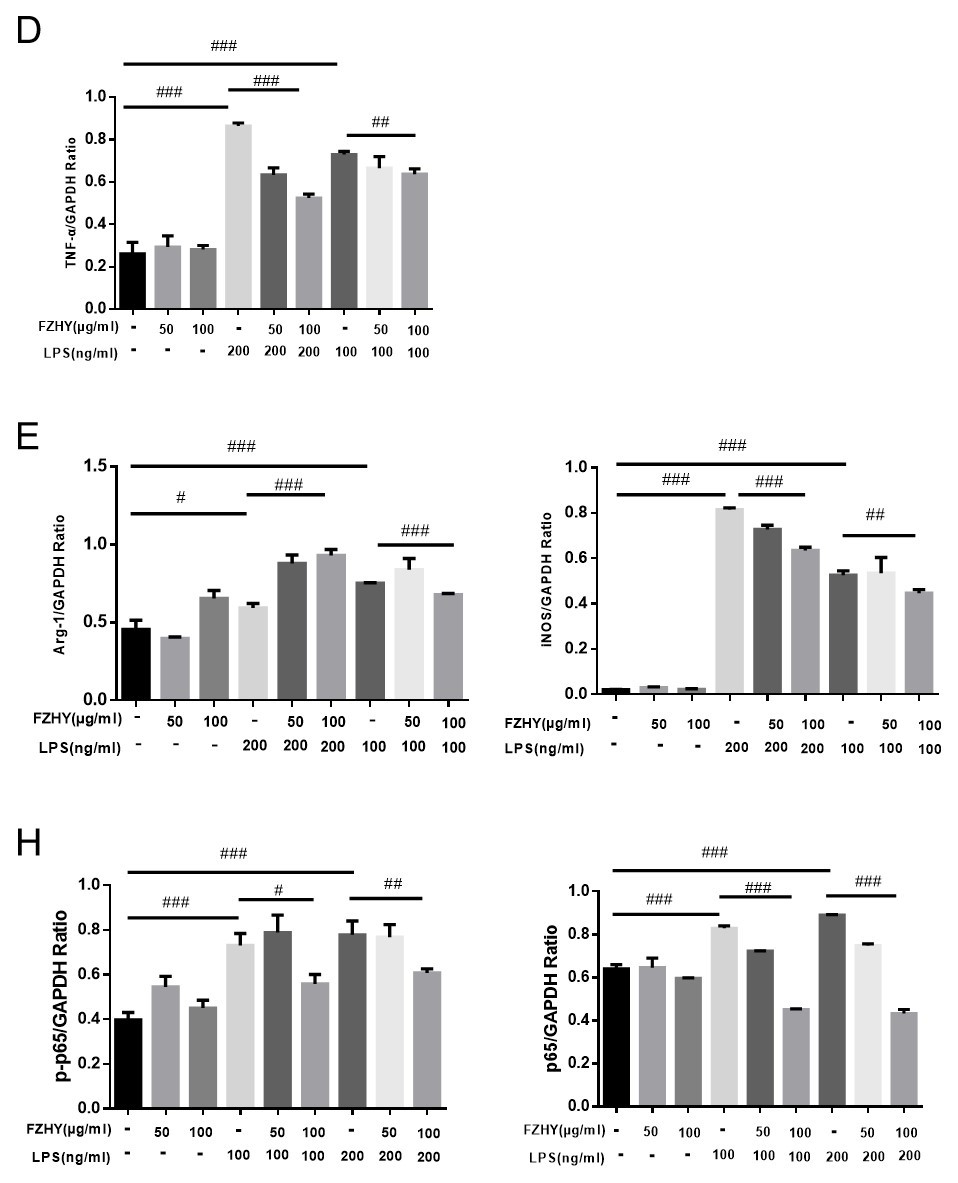

Supplement: Supplementary file 5 [file Image1.jpg]
